# The subthreshold-active K_V_7 current regulates neurotransmission by limiting spike-induced Ca^2+^ influx in hippocampal mossy fiber synaptic terminals

**DOI:** 10.1038/s42003-019-0408-4

**Published:** 2019-04-26

**Authors:** Katiuscia Martinello, Elisabetta Giacalone, Michele Migliore, David A. Brown, Mala M. Shah

**Affiliations:** 1 UCL School of Pharmacy University College London, London, WC1N 1AX UK; 2 Institute of Biophysics, National Research Council, 90146 Palermo, Italy; 3 Department of Neuroscience, Physiology and Pharmacology, University College London, London, WC1E 6BT UK

**Keywords:** Ion channels in the nervous system, Cellular neuroscience

## Abstract

Little is known about the properties and function of ion channels that affect synaptic terminal-resting properties. One particular subthreshold-active ion channel, the Kv7 potassium channel, is highly localized to axons, but its role in regulating synaptic terminal intrinsic excitability and release is largely unexplored. Using electrophysiological recordings together with computational modeling, we found that the K_V_7 current was active at rest in adult hippocampal mossy fiber synaptic terminals and enhanced their membrane conductance. The current also restrained action potential-induced Ca^2+^ influx via N- and P/Q-type Ca^2+^ channels in boutons. This was associated with a substantial reduction in the spike half-width and afterdepolarization following presynaptic spikes. Further, by constraining spike-induced Ca^2+^ influx, the presynaptic K_V_7 current decreased neurotransmission onto CA3 pyramidal neurons and short-term synaptic plasticity at the mossy fiber–CA3 synapse. This is a distinctive mechanism by which K_V_7 channels influence hippocampal neuronal excitability and synaptic plasticity.

## Abstract (web summary)

Martinello et al. show that the KV7 K^+^ channels, active at rest, diminish action potential-induced Ca^2+^ influx into hippocampal mossy fiber boutons. This reduction alters presynaptic spikes, reduces neurotransmitter release, and decreases short-term synaptic plasticity, highlighting a role for these channels in hippocampal CA3 neuronal excitability.

## Introduction

Neurotransmitter release from synaptic terminals is the predominant mechanism for information transfer between neurons and has a fundamental role in processes such as synaptic plasticity^1–3^. Diverse synaptic terminals express the high voltage-activated K_V_1 and K_V_3 channels that are activated during the repolarization phase of the presynaptic action potential. These K^+^ channels regulate the action potential width and, consequently, presynaptic Ca^2+^ influx and neurotransmission^4–16^. There are, though, K^+^ channels that activate at rest (i.e., at subthreshold potentials)^17^. Much less is known about the properties and function of these channels in synaptic terminals.

The K_V_7 channels activate at voltages positive to − 70 mV in many neurons and form an inhibitory current at rest^17–19^. There are 5 K_V_7 subunits, of which K_V_7.2–K_V_7.5 subunits are neuronal. The K_V_7.2 and K_V_7.3 subunits contain an ankyrin G-binding motif, so they are expressed at the axon initial segment and nodes of Ranvier where they regulate the action potential threshold and propagation^17,18,20–23^. Interestingly, K_V_7.5 channels are present in the giant synaptic terminals of Calyx of Held, where they influence the resting membrane potential (RMP)^24^. Immunohistochemical evidence also suggests that K_V_7.5 subunits are situated in GABAergic, but not glutamatergic, synaptic terminals in the hippocampus^25^. In contrast, immunohistochemistry suggests that K_V_7.2 subunits are expressed throughout hippocampal mossy fibers^26–28^. However, it is not known if hippocampal mossy fiber boutons have a K_V_7 current or whether this affects their local intrinsic excitability and neurotransmitter release. It is important to investigate this as the mossy fiber giant bouton-CA3 pyramid synapse has a critical role in processes such as learning and memory and in pathophysiological disorders such as epilepsy^29–33^.

Here, we show that adult mossy fiber boutons possess a K_V_7 current that is active at rest. This had little effect on the bouton RMP. The current, though, altered the intrinsic excitability of boutons by decreasing the membrane resistance at voltages positive to rest. The current also limited action potential-induced Ca^2+^ influx via voltage-gated N- and P/Q-type Ca^2+^ channels. This was coupled with a reduction in the presynaptic spike half-width and an afterdepolarization that follows presynaptic spikes in these boutons. Further, by limiting action potential-induced Ca^2+^ influx, K_V_7 channels restricted neurotransmitter release and short-term synaptic plasticity onto CA3 pyramidal neurons. This is a unique mechanism by which presynaptic K_V_7 channels affect local excitability within adult hippocampal synaptic terminals and regulate neurotransmission. This might be an important means by which K_V_7 channels contribute towards influencing neural network rhythms and maintaining network excitability in the hippocampus^34–36^.

## Results

### K_V_7 currents in mature mossy fiber boutons

**Fig. 1:**
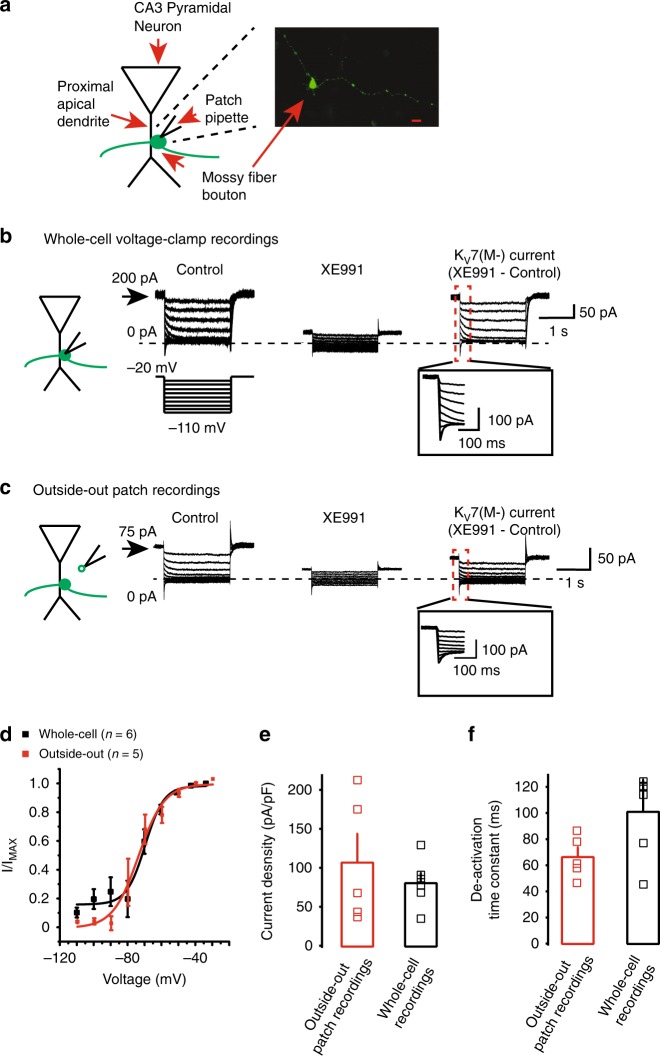
The K_V_7 current is localized in mossy fiber boutons. **a** Schematic showing that mossy fiber boutons from which electrophysiological recordings were obtained were situated near or on the proximal dendrites of CA3 pyramidal neurons. The insert shows a confocal image of a mossy fiber bouton that had been recorded from, filled with neurobiotin and stained with streptavidin Alexa Fluor 488 conjugated antibodies. The scale bar corresponds to 2 μm. **b**, **c** Example whole-cell and outside-out voltage-clamp recordings of the K_V_7 current, respectively. The bouton or patch was held at −20 mV and a series of hyperpolarizing, 2 s long steps were applied as per schematic in the absence (control) and presence of 3 μm XE991. The current in the presence of XE991 was subtracted from that recorded in the absence to obtain the K_V_7 current. The de-activation rates of this current are shown on an expanded time scale in the inset. The scale associated with the first trace applies to all traces within the panel. The outward holding current at −20 mV under control conditions is also shown. **d** The activation curves of the K_V_7 current measured under either whole-cell or outside-out patch voltage-clamp conditions. **e**, **f** The average (bars) and individual (open squares) current (I) density and de-activation time constants (*τ*) in five outside-out patches and six whole-cell recordings from mossy fiber boutons, respectively. The capacitance for calculating the current density was obtained using our multiclamp amplifier. The *τ* values were measured for currents elicited by a hyperpolarizing step to −50 mV from a holding potential of −20 mV

As mossy fiber boutons that synapse onto CA3 proximal apical dendrites have large diameters (2–5 μm, Fig. 1a)^10,31,32,37^, we made electrophysiological recordings from these present in hippocampal slices obtained from mature rats. The tracer, neurobiotin, was included and post-hoc morphological analysis was performed to positively identify boutons (Fig. 1a). To record the K_V_7 current, the classical de-activation protocol^38–40^ was applied under whole-cell voltage-clamp condition in the absence and presence of the specific, irreversible, pharmacological K_V_7 channel inhibitor, XE991 (3 μm, a concentration that inhibits > 95% of the current^41^) (Fig. 1b). This revealed slow de-activating currents that reversed at ~ − 90 mV (i.e., near the K^+^ reversal potential, Fig. 1b). The currents were stable for at least 20 min, with minimal rundown and were maximally inhibited by 20 min bath application of XE991 (Fig. 1b), suggesting that the K_V_7 current was present in mossy fiber boutons.

To determine whether the K_V_7 currents recorded under whole-cell voltage-clamp conditions were generated, at least partly, in the bouton per se, we made outside-out patch recordings from the boutons (Fig. 1c). Slow de-activating currents with comparable properties to those obtained under whole-cell voltage-clamp experiments were present in outside-out patches too. These were fully inhibited by XE991 (Fig. 1c). The half voltage-activation (V_1/2_) and slopes of the activation curves from whole-cell and outside-out patch configurations were similar (whole-cell and outside-out patch V_1/2_ = −69.6 ± 1.6 mV (*n* = 6) and −73.9 ± 1.8 mV (*n* = 5), respectively; whole-cell and outside-out patch activation curve slopes = 5.5 ± 1.5 (*n* = 6) and 7.6 ± 1.7 (*n* = 5), respectively; Fig. 1d; see Methods). The current density and de-activation time constants measured at − 50 mV were comparable in both configurations (Fig. 1e, f). Given the remarkable similarities in K_V_7 current densities and biophysical properties between outside-out patch and whole-cell voltage-clamp configurations, our results strongly suggest that K_V_7 channels are located on mature mossy fiber boutons synapsing onto CA3 pyramidal neurons.

### K_V_7 currents limit the membrane resistance and excitability

**Fig. 2:**
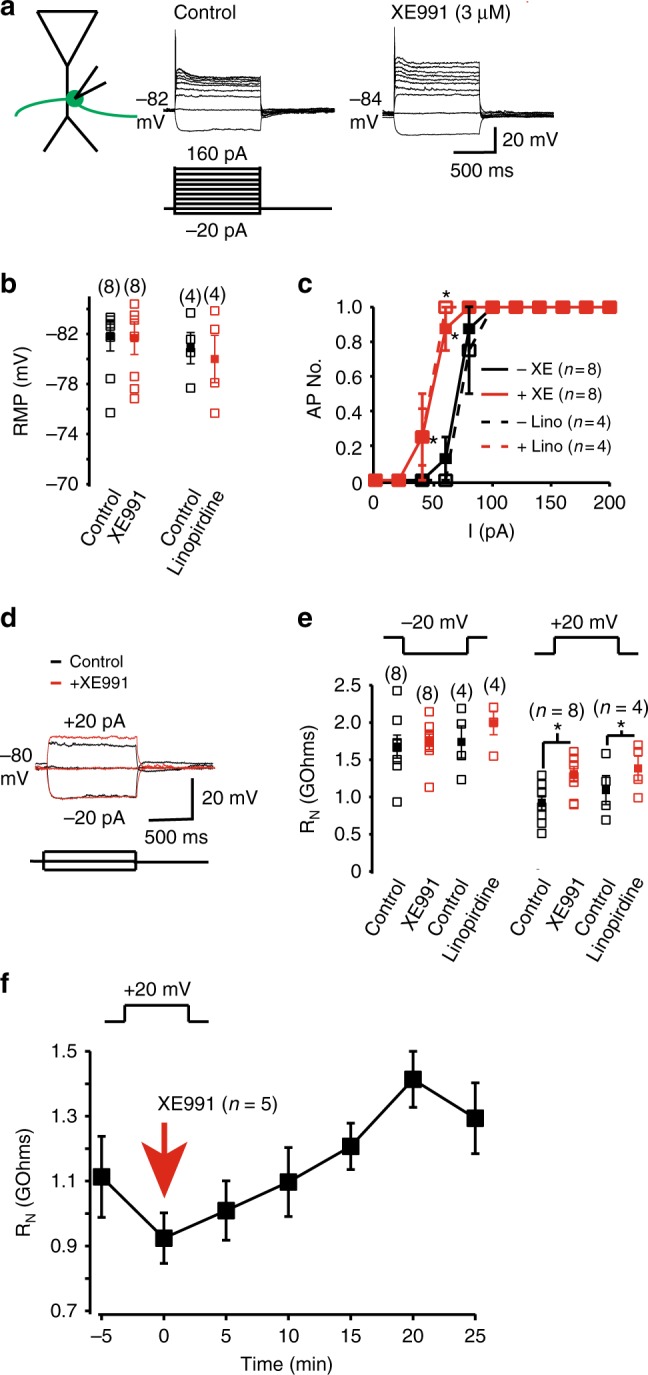
K_V_7 currents regulate the intrinsic excitability of mossy fiber boutons. **a** Representative whole-cell current-clamp recordings generated when a series of hyperpolarizing and depolarizing current pulses were applied to a mossy fiber bouton before and after application of 3 μM XE991 at the normal resting membrane potential (RMP, indicated adjacent to the traces). The scale shown applies to both traces. **b**, **c** Graphs depicting the individual (open square) and mean (filled square) RMP as well as the average numbers of action potentials recorded from boutons in response to current (I) pulses in the absence and presence of XE991 or linopirdine (10 μM) respectively. The numbers of observations are indicated in parenthesis. **d** Example recordings obtained when 20 pA, 1 s hyperpolarizing and depolarizing square current pulses were applied at a fixed potential of − 80 mV in the absence and presence of XE991. **e** The individual (open square) and mean (filled square) input resistance (*R*_N_) measured using 20 pA hyperpolarizing and depolarizing current pulses at − 80 mV with and without XE991 or linopirdine. The numbers of observations are indicated in parenthesis. **f** The time course of the average effects of XE991 on the input resistance measured using a 20 mV depolarizing step in five independent mossy fiber terminals. In all panels, asterisks signify *p* < 0.05 when compared with appropriate controls

To ascertain whether K_V_7 channels affect mossy fiber bouton intrinsic excitability, we made whole-cell current-clamp recordings from boutons in the presence of glutamate and GABA receptor blockers (see Methods) in the absence and presence of XE991 (3 μM). The boutons had an average RMP of − 81.9 ± 1.2 mV (*n* = 8), which was unaffected by 20 min bath application of XE991 (average RMP with XE991 = −81.7 ± 1.3 mV (*n* = 8, *p* = 0.83, two-tailed paired *t* test); Fig. 2a, b). Similar findings were also obtained with 20 min application of a second K_V_7 channel inhibitor, linopirdine (10 μM)^41^ (Fig. 2b). Given that our data (Fig. 1d) suggests that ~ 20% of the K_V_7 current is active at −80 mV, this implies that other ion channels, such as the inward rectifier potassium channels^42,43^ and twin-pore potassium channels, have a larger influence on the RMP in mossy fiber boutons.

Depolarizing current pulses of varying magnitudes resulted in only single action potentials in boutons (Fig. 2a). The inability of more than one spike to be initiated with different magnitudes and sustained depolarization is probably owing to the specialized properties of the mossy fiber bouton Na^+^ and other K^+^ (e.g., K_V_1) channels^14,44^. In the presence of either XE991 or linopirdine, though, significantly less depolarizing current was required for action potential initiation (Fig. 2a–c). Thus, K_V_7 channels enhance the rheobase for spike generation in mossy fiber boutons.

If K_V_7 channels do not affect the RMP in mossy fiber boutons, how might they affect the ability for depolarizing pulses to result in spike generation here? In many other neurons, the inhibitory current formed by K_V_7 channels at subthreshold potentials limits the amount of depolarization with given current injections (i.e., decreases the input resistance (*R*_N_)), resulting in a larger rheobase required to elicit action potentials. Thus, we examined whether these channels modified *R*_N_ in mossy fiber boutons too. To test this, we injected small hyperpolarizing and depolarizing subthreshold current pulses at a fixed potential of − 80 mV (i.e., near the bouton RMP) in the absence and presence of XE991 or linopirdine (Fig. 2d). *R*_N_ measured at potentials above −80 mV, but not at hyperpolarizing potentials, was significantly greater in the presence of XE991 or linopirdine (Fig. 2d, e). This effect was time-dependent, peaking ~20 min after application of XE991 (Fig. 2f). As mossy fiber boutons are electronically compact^14,45^, the effect on *R*_N_ is likely to be owing to local K_V_7 channels in the mossy fiber bouton. Indeed, in the granule cell somata where K_V_7 channels are not present, K_V_7 channel inhibitors have little effect on *R*_N_^40^. Hence, in mossy fiber boutons, K_V_7 channels generate an inhibitory current that restricts R_N_ at positive potentials to rest and thereby, limits the number of presynaptic spikes elicited by depolarization.

### K_V_7 currents reduce spike width and afterdepolarization

**Fig. 3:**
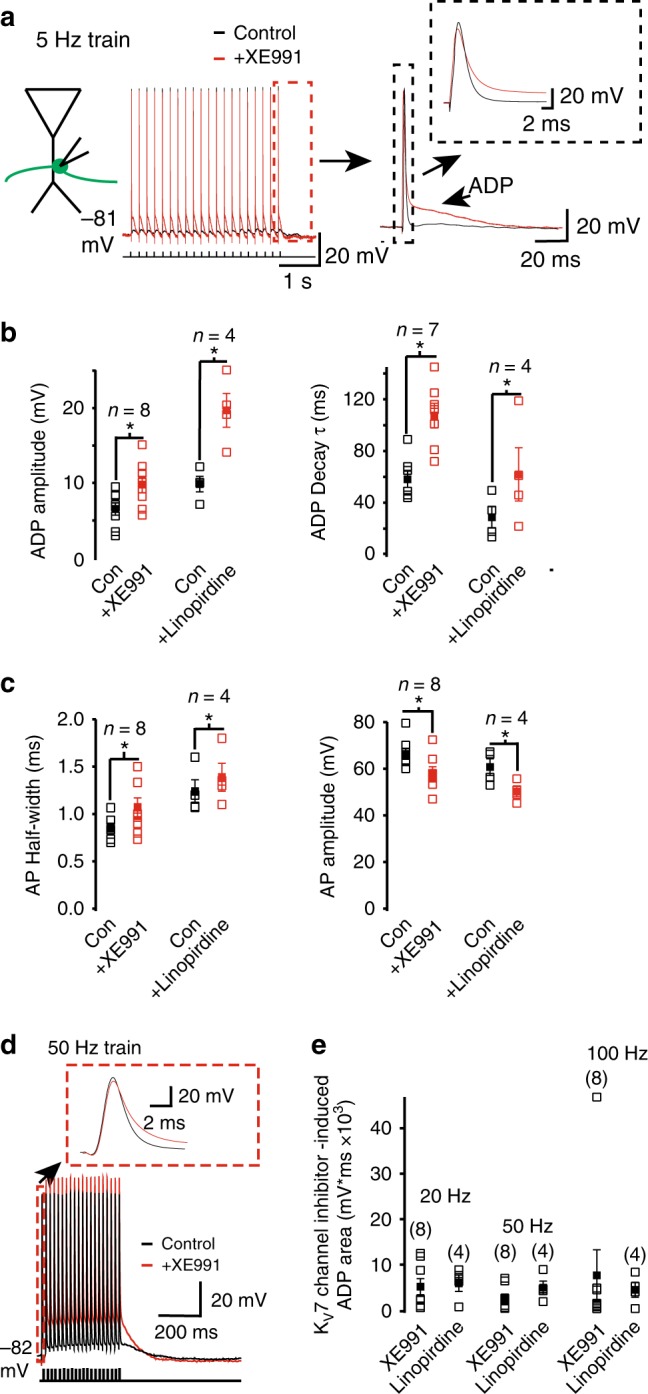
K_V_7 currents constrain the spike width and ADP following action potentials in mossy fiber boutons. **a** Example 5 Hz train of action potentials obtained under whole-cell current-clamp conditions before and after XE991 application at RMP (indicated adjacent to the trace). Each action potential was elicited by applying a 0.1 ms depolarizing current injection. The last action potential of the train with the associated afterdepolarization (ADP) is shown on a larger scale on the right. The inset shows the action potential shape with and without XE991. **b**, **c** Graphs depicting the ADP amplitude and decay time constant (*τ*) as well as the action potential (AP) half-width and amplitude respectively in the absence (con) and presence of either XE991 or linopirdine. Open and filled squares represent the individual and mean values respectively. The data shown are of the 1st action potential and ADP kinetics obtained for a train at 5 Hz. The numbers of observations are indicated above each set of bars. **d** Representative traces showing trains of 20 action potentials elicited at 50 Hz in the absence (control) and presence of XE991. The first action potential is shown on an expanded scale in the inset. **e** The individual (open square) and mean (filled square) K_V_7 channel inhibitor-induced ADP area associated with 20, 50, and 100 Hz trains of 20 action potentials. The numbers of observations are indicated in parenthesis. In all graphs, significance at *p* < 0.05 when compared with the appropriate control is indicated by asterisks (*)

In most neurons, single or trains of spikes invade synaptic terminals, leading to neurotransmitter release^1–3^. Thus, we injected very short current pulses (0.1 ms) to evoke single or trains of action potentials at various frequencies (1, 5, 20, 50, and 100 Hz; Fig. 3). Each spike was followed by a small intrinsically generated afterdepolarization (ADP) (Fig. 3a). This ADP duration was ≥ 150 ms, such that with spike trains generated at ≥ 20 Hz, the spikes did not initiate during the ADP (Fig. 3a). Inhibition of K_V_7 currents by XE991 or linopirdine enhanced the ADP amplitude and decay time constant following a single action potential to a similar extent irrespective of the frequency of the spike train or the position of the action potential within the train (Fig. 3b; Supplementary Table 1). The spike width in the presence of either XE991 or linopirdine was also broader than under control conditions (Fig. 3c). Further, the spike amplitudes were smaller in the presence of K_V_7 channel inhibitors compared with controls (Fig. 3c; Supplementary Table 2).

At frequencies of ≤ 20 Hz, under control conditions, each subsequent spike in a train occurred during the ADP generated by the preceding action potential, resulting in a summating ADP (Fig. 3d, e). The area under the ADP was much greater after application of XE991 or linopirdine than under control conditions (Fig. 3d, e). As K_V_7 channel inhibition at the granule cell axon initial segment did not result in enhancement of an ADP^40,46^, it suggests that these channels differentially regulate intrinsic excitability in granule cell axon subcompartments.

### The K_V_7 conductance limits spike-induced Ca^2+^ concentration

**Fig. 4:**
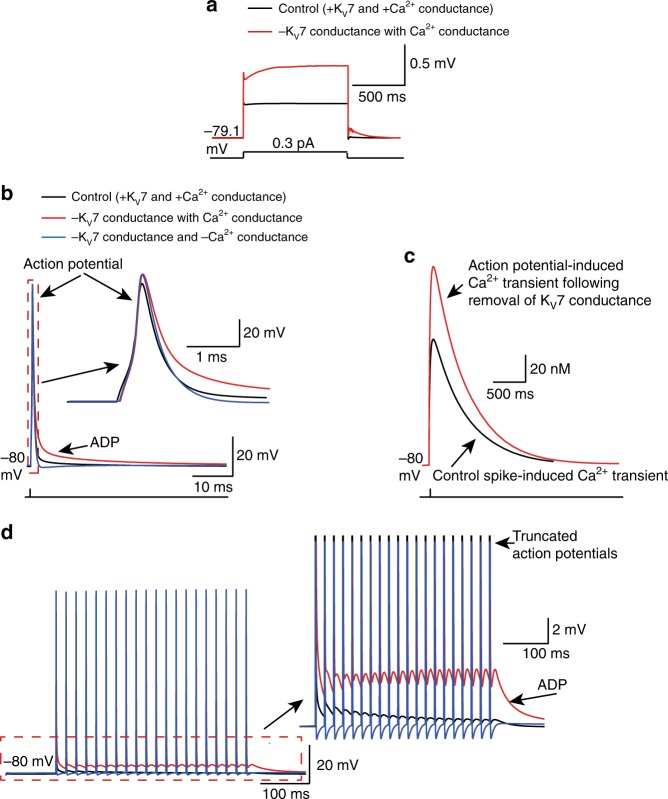
Computational model illustrating that the spike half-width and ADP proceeding spikes in the absence of a K_V_7 conductance is owing to enhanced intracellular Ca^2+^. **a** Simulation showing the voltage change in response to a subthreshold 1 s square depolarization in the absence and presence of a K_V_7 conductance. The traces under the two conditions have been superimposed. **b** Simulated single action potentials elicited by a short depolarizing step with and without a K_V_7 conductance. In addition, a single action potential in the absence of both the K_V_7 and Ca^2+^ conductances was also generated (blue). The inset shows the individual action potentials on a larger scale. **c** Intracellular Ca^2+^ changes in response to the same stimulus that elicited an action potential with (black) and without (red) the K_V_7 conductance. **d** A train of action potential waveforms at 50 Hz when the K_V_7 conductance was intact (black), following removal of the K_V_7 conductance in the presence of the Ca^2+^ conductance (red) or after removal of both the K_V_7 and Ca^2+^ conductances (blue). The inset shows the ADP following action potentials under the three conditions on an expanded scale

To further understand the cellular mechanisms by which K_V_7 channels might restrict the spike width and the ADP amplitude following spikes, we generated a single compartment model consisting predominantly of the K_V_7 conductance with our biophysical characteristics (Fig. 1), an inward rectifier type K^+^ conductance, the ‘A’-type (K_V_1) conductance, delayed rectified type K^+^ conductance, Na^+^ conductance and a Ca^2+^ conductance (see Supp. Table 3 and Methods). The RMP under these conditions was −79.1 and −78.6 mV upon removal of the K_V_7 conductance. This small (0.5 mV) depolarization caused by loss of the K_V_7 conductance is within experimental error and would not have been detected in experiments. Ablation of the K_V_7 conductance, though, enhanced the *R*_N_ from 1.00 GΩ to 2.33 GΩ when measured using depolarizing subthreshold current pulses (Fig. 4a). These findings are consistent with experimental observations (Fig. 2).

Next, we simulated an action potential with and without the K_V_7 conductance (Fig. 4b). In agreement with our experimental observations (Fig. 3, Supplementary Table 2), the spike width broadened from 0.55 ms to 0.75 ms upon removal of the K_V_7 conductance. In the absence of the K_V_7 conductance (Fig. 4b), the spike amplitude was higher than controls. The model suggested that a reduction in spike amplitude, consistent with experiments, could be produced by reducing the Na^+^ conductance by 20% (Supplementary Fig. 1).

We also modeled the changes in Ca^2+^ concentration induced by a single spike (Fig. 4c). Consistent with previous studies^47–49^, the simulations predicted that with a K_V_7 conductance (i.e., under control conditions), an action potential caused the intracellular Ca^2+^ concentration to increase by 100 nM (Fig. 4c). Removal of the K_V_7 conductance induced a further rise in intracellular Ca^2+^ by 60 nM (Fig. 4c). Given that the Ca^2+^ conductance is activated during a spike^50,51^, the model suggests that the rise in intracellular Ca^2+^ during the spike would have contributed to the spike broadening in the absence of the K_V_7 conductance. Indeed, the spike width in our simulations without the K_V_7 and Ca^2+^ conductance was very similar to control conditions (i.e., when both conductances were active; Fig. 4b).

Removing the K_V_7 conductance also resulted in the generation of an ADP with a decay time constant of 22.9 ms (Fig. 4b). In agreement with our experimental observations, a train of 20 action potentials at a frequency of 50 Hz resulted in successive action potentials being initiated near the peak of the ADP generated by the previous spike (Fig. 4d). The enhanced ADP following spikes in the absence of the K_V_7 conductance was abolished when the Ca^2+^ conductance was removed (Fig. 4b, d). These findings imply that the K_V_7 current in boutons serves to suppress a rise in intracellular Ca^2+^ concentration during an action potential and regulates the spike width and generation of an ADP succeeding the presynaptic spike.

### BAPTA prevents K_V_7 inhibitor effects on spike with and ADP

**Fig. 5:**
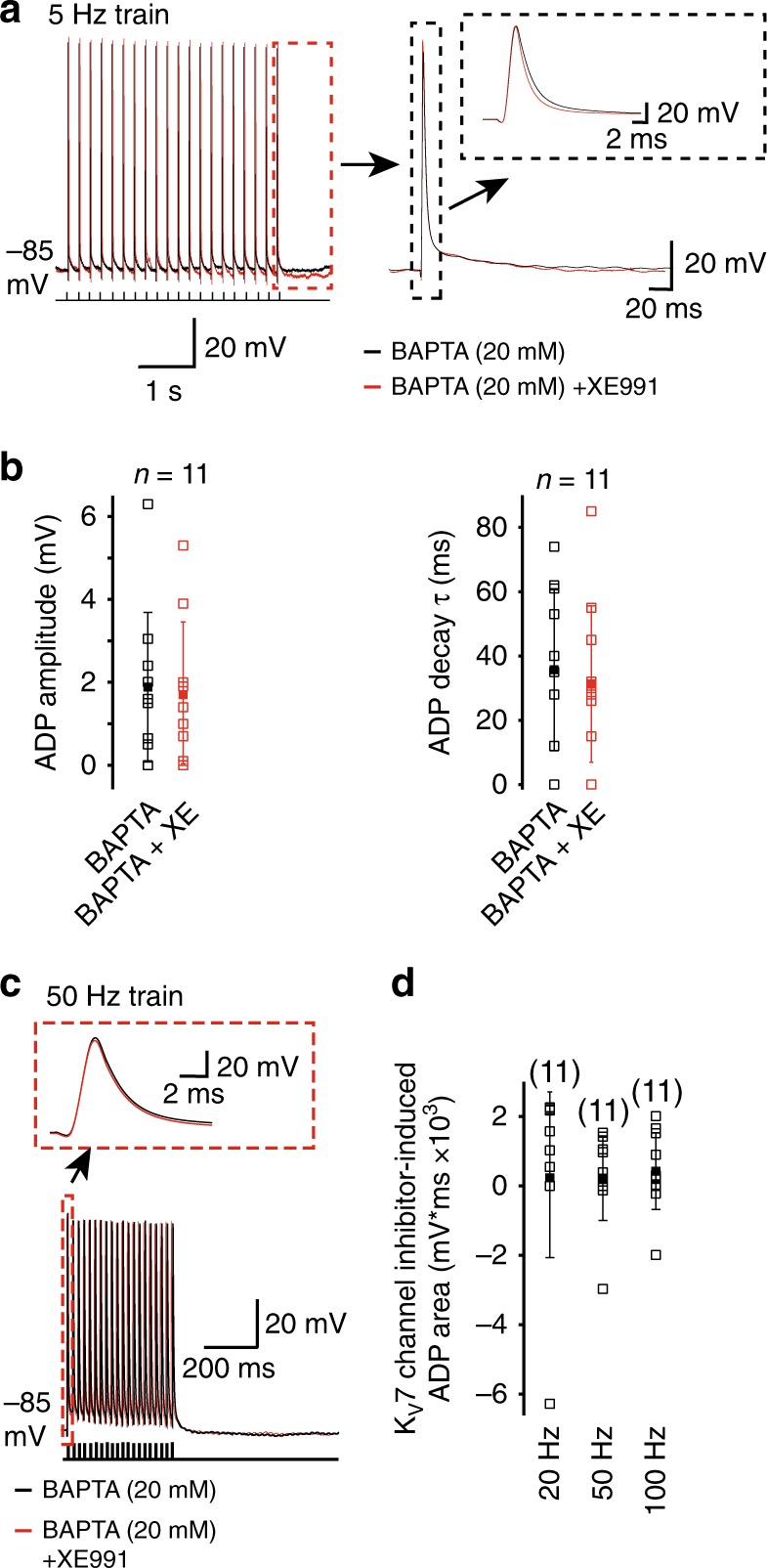
The Ca^2+^ chelator, BAPTA, prevents K_V_7 current inhibition-induced spike broadening and enhanced ADP following action potentials. **a** Example 5 Hz action potential trains when 20 mm BAPTA was included in the patch pipette in the absence and presence of 3 μm XE991. The last action potential with the associated ADP is shown on an expanded time scale. The spike itself is shown in the inset. **b** Graphs depicting the individual (open squares) and mean (filled square) values for the ADP amplitude and decay time constant (*τ*) before and after application of XE991 when BAPTA was included in the patch pipette. **c** Representative 50 Hz action potential trains in the absence and presence of XE991 with 20 mm BAPTA in the patch pipette. The first action potential is shown on an expanded time scale in the inset. **d** The individual (open square) and mean (filled square) area underlying the K_V_7 current inhibition-induced ADP generated with trains of action potentials at 20, 50, and 100 Hz when BAPTA was incorporated in the patch pipette solution

To test whether the effects of K_V_7 channel inhibition on the spike half-width and ADP is owing to a rise in intracellular Ca^2+^, we included the Ca^2+^ chelator, BAPTA (10 mM or 20 mM) in the intracellular patch pipette (see Methods). The findings with 10 mM and 20 mM BAPTA were no different and have been grouped together. Full dialysis of BAPTA into boutons occurred within 5 min. Stable recordings could be obtained for at least 30 min with BAPTA in the intracellular solution. Under these conditions, 20 min application of XE991 had little effect on the single action potential half-width or amplitude (Fig. 5a, Supplementary Table 1).

In addition, XE991 had little effect on the ADP amplitude or decay time constant elicited by single action potentials at either 1 or 5 Hz when BAPTA was included in the patch pipette (Fig. 5, Supplementary Table 1). This was independent of the spike frequency or the position of the spike within the train (Supplementary Table 1). Further, there was no increase in the ADP generated during a train of action potentials elicited at frequencies of 20, 50, or 100 Hz in the presence of XE991 when BAPTA was present in the patch pipette solution (Fig. 5). Thus, these findings further support the suggestion that K_V_7 channel inhibition in mossy fiber boutons induces a rise in intracellular Ca^2+^ that leads to spike broadening and an enhanced ADP following presynaptic spikes.

### K_V_7 currents limit Ca^2+^ influx to affect spike width and ADP

**Fig. 6:**
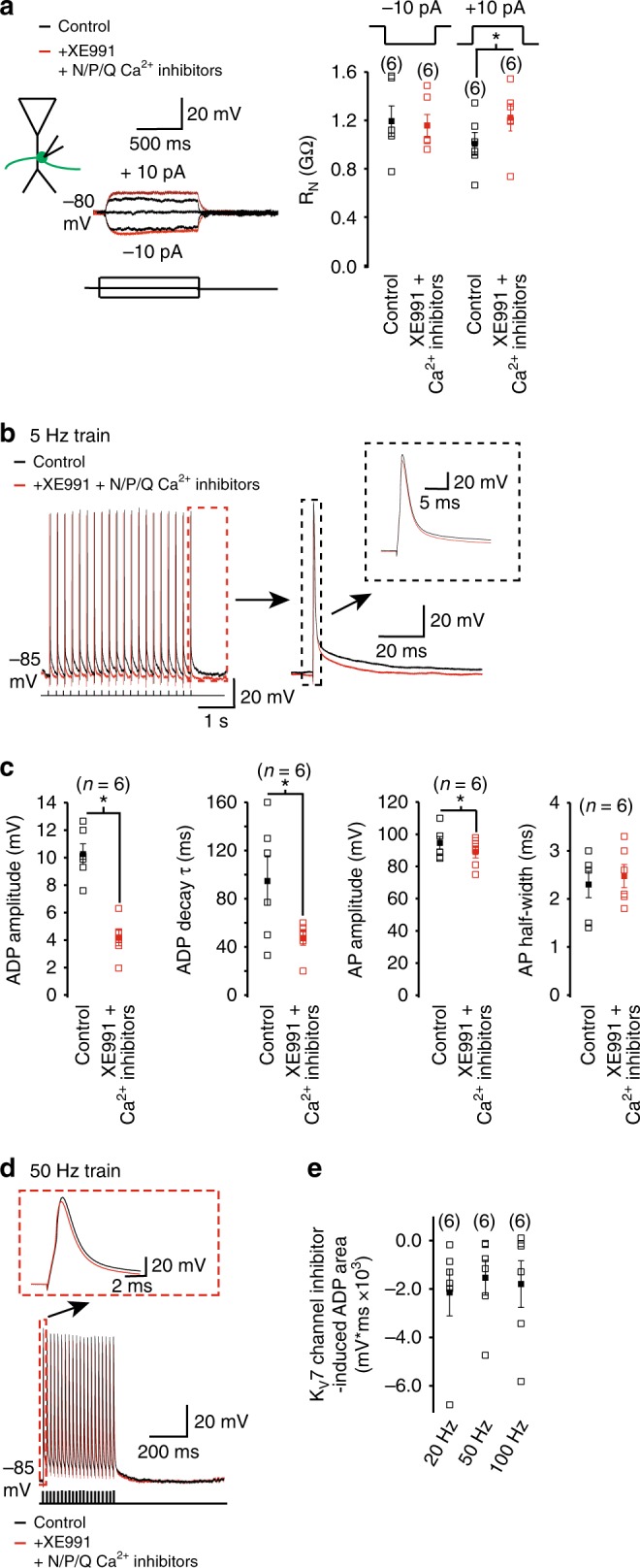
K_V_7 currents limit Ca^2+^ influx partly via N- and P/Q-type Ca^2+^ channels to reduce the action potential half-width and ADP following spikes in mossy fiber boutons. **a** Representative recordings obtained with subthreshold hyperpolarizing and depolarizing current pulses before and after co-application of XE991 and N- and P/Q-type Ca^2+^ channel inhibitors. The individual (open squares) and average (filled squares) input resistance (*R*_N_) values obtained using this protocol are shown in the graph. The numbers of observations per group are depicted above each bar. **b**, **d** Typical 5 and 50 Hz trains of action potentials under control conditions and following application of XE991 and N- and P/Q-type Ca^2+^ channel inhibitors. The first and last action potential in the 50 and 5 Hz trains, respectively, are shown in the insets. The ADP associated with the last action potential in the 5 Hz train is also illustrated on a larger scale in the inset in **b**. **c** The individual (open squares) and mean (filled squares) peak ADP amplitude and decay time constant (*τ*)) and the action potential (AP) amplitude and half-width associated with the first action potential of a 5 Hz train of action potentials in the absence and presence of XE991 and N- and P/Q-type Ca^2+^ channel inhibitors. **e** The individual neuron (open squares) and average (filled squares) area of the K_V_7 channel inhibitor-induced ADP with 20, 50, and 100 Hz trains of action potentials in the presence of N- and P/Q-type Ca^2+^ channel inhibitors. The numbers of observations for each group are shown in parenthesis. Asterisks (*) denote significance at *p* < 0.05 when compared with appropriate controls

Next, we asked what might be the source of the rise in intracellular Ca^2+^ in the absence of K_V_7 channels? Mossy fiber boutons express predominantly P/Q-type voltage-gated Ca^2+^ channels as well as N- and R-type Ca^2+^ channels^51^. The N- and P/Q-type channels are activated during presynaptic spikes in mossy fiber boutons^51^. We, therefore, investigated whether Ca^2+^ influx via these channels might contribute towards the spike broadening and increase in ADP amplitude and decay time constant caused by K_V_7 channel inhibition. For this, we tested the effects of co-application of 3 μm XE991 and the selective N- and P/Q-type Ca^2+^ channel inhibitors, 100 nm ω-conotoxin GVIA and 100 nM ω-agatoxin IVA. With these inhibitors, the *R*_N_ measured by applying depolarizing, but not hyperpolarizing, subthreshold steps was still enhanced compared with that prior to application of the compounds (Fig. 6a).

We then elicited trains of action potentials at different frequencies in the absence and presence of XE991, ω-conotoxin GVIA and ω-agatoxin IVA. Whereas the spike amplitude was still significantly reduced by application of the inhibitors (Fig. 6b, c, Supplementary Table 2), there was little difference in spike width with and without the inhibitors (Fig. 6b, c, Supplementary Table 2). Further, we found that the ADP amplitude and decay time constant following spikes occurring at 1 Hz and 5 Hz was significantly reduced in the presence of XE991 and N- and P/Q-type Ca^2+^ channel inhibitors compared with that controls (Fig. 6b, c, Supplementary Table 1).

As previously found (Fig. 3d), action potential trains at ≤ 20 Hz resulted in spikes being generated on the ADP under control conditions (Fig. 6d). Co-application of XE991 and N- and P/Q-type Ca^2+^ channel inhibitors resulted in a reduction of the ADP area compared with control conditions such that spikes now occurred at the normal RMP (i.e., at the baseline; Fig. 6d, e). Altogether, these findings robustly support the notion that the K_V_7 current reduces Ca^2+^ influx via N- type and P/Q-type Ca^2+^ channels and, thereby, regulates the spike width and ADP generated following spikes in mossy fiber boutons.

### K_V_7 channels reduce neurotransmission onto CA3 neurons

**Fig. 7:**
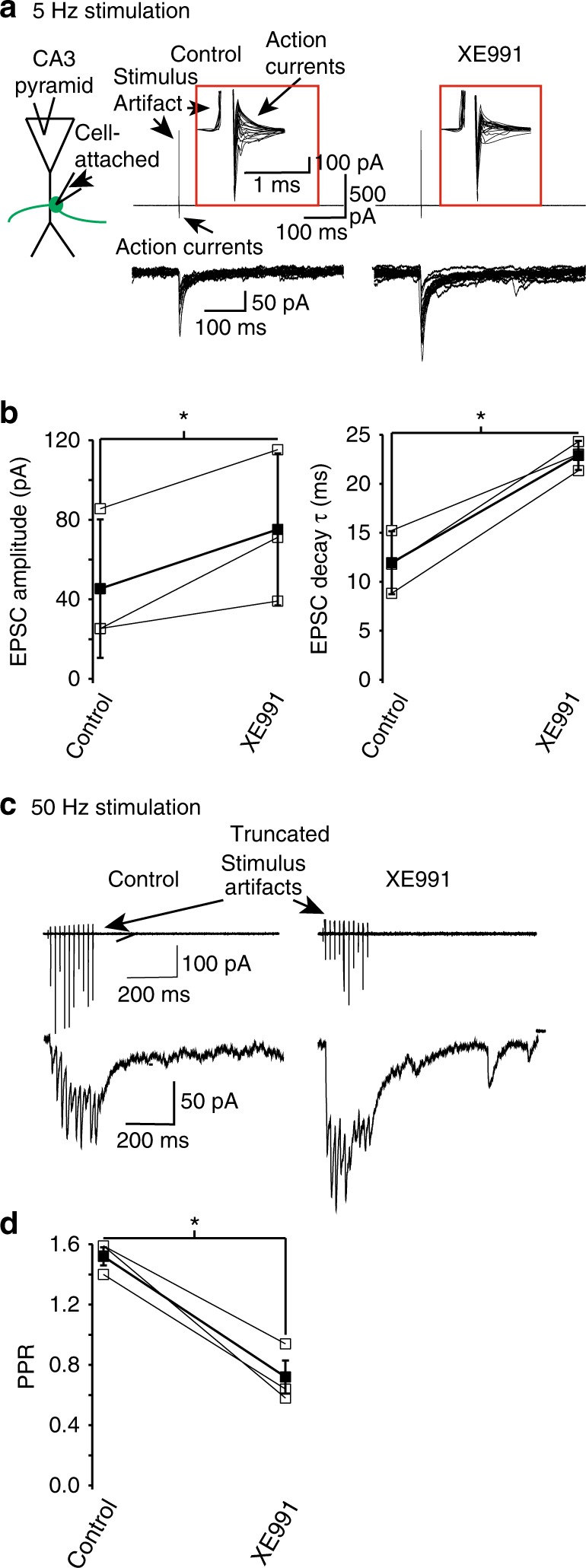
Presynaptic K_V_7 currents reduce neurotransmitter release from mossy fiber boutons onto CA3 pyramidal neurons. **a** Schematic illustrating the paired recording configuration obtained from CA3 pyramidal somata and a mossy fiber bouton synapsing onto its proximal apical dendrite. Also shown are typical single EPSCs elicited in CA3 pyramidal neurons at a fixed potential of − 70 mV when action currents at a frequency of 5 Hz were evoked in mossy fiber boutons that contacted their proximal apical dendrites. The EPSCs were obtained in the absence and presence of 3 μm XE991. The action currents are shown on an expanded scale in the inset (red box). The scales associated with the first pair of traces apply to both sets of traces. **b** The average (filled squares) EPSC amplitudes and decay time constants (*τ*) when evoked at 5 Hz in three different mossy fiber bouton–CA3 pyramid pairs before and after XE991 application. The open squares represent the mean of 10 EPSC amplitudes and decay time constants obtained from individual mossy fiber bouton–CA3 pyramid pairs. **c** Example recordings of 10 EPSCs evoked in a CA3 pyramidal cell by a train of 50 Hz action currents in a mossy fiber bouton synapsing onto the neuron under control conditions and following XE991 application. The scales shown on the first pair of traces apply to the second. The amplitudes of the first and second EPSCs in the train were measured to obtain the paired pulse ratio (PPR). **d** The average (filled square) and individual PPRs (open squares) from three bouton–CA3 neuron pairs without and with XE991. Asterisks (*) denote significance at *p* < 0.05 when compared with appropriate controls

Given that our findings suggest that K_V_7 channels limit Ca^2+^ influx via N- and P/Q-type Ca^2+^ channels, which regulate synaptic release from mossy fiber boutons^10,51–53^, we hypothesized that K_V_7 channel inhibition should enhance neurotransmitter release from mossy fiber boutons onto CA3 pyramidal neurons. To investigate this, we obtained cell-attached recordings from a mossy fiber bouton and whole-cell voltage-clamp recordings from the CA3 pyramidal neuron whose proximal apical dendrite the bouton was next to in the absence of glutamate and GABA receptor blockers (see Fig. 7a^45^). As CA3 pyramidal neurons express K_V_7 channels postsynaptically^54^, we replaced intracellular K^+^ in the CA3 pyramidal neuron with Cs^+^ to inhibit these channels (see Methods). We also included QX314 bromide in the CA3 pyramidal recording solution to inhibit Na^+^ channels. As dentate–gyrus granule cells are most likely to fire action potentials phase-locked to theta or gamma rhythms in vivo^55^, we initially induced action currents at 5 Hz (i.e., theta frequency) in the bouton as in ref. ^56^ (Fig. 7a). This resulted in mono-synaptic excitatory post-synaptic currents (EPSCs) in the post-synaptic CA3 neuron with a very low failure rate (% failure = 8.33 ± 10.2%, *n* = 3, Fig. 7b). Subsequent application of XE991 (3 μm) enhanced the EPSC amplitude and decay time constant (Fig. 7a, b), strongly suggesting that presynaptic K_V_7 channels constrain neurotransmitter release from mossy fiber boutons.

As spike trains in granule cells at gamma frequency have a higher probability of initiating action potentials in CA3 pyramidal neurons during certain types of behavior^45,57^, we also investigated how presynaptic K_V_7 channels would affect neurotransmitter release when the spike frequency was 50 Hz. For this, we elicited 10 action currents at 50 Hz in boutons (Fig. 7c). As has been previously described^45^, this resulted in paired pulse facilitation of EPSCs (Fig. 7c, d). Bath application of XE991 significantly reduced the paired pulse ratio of evoked EPSCs (Fig. 7c, d), indicating that presynaptic K_V_7 channels restrict neurotransmitter release from mossy fiber boutons independently of spike frequency. Further, these findings robustly suggest that presynaptic K_V_7 channels affect short-term synaptic plasticity at this hippocampal synapse.

## Discussion

Here, we show that mossy fiber boutons possess a K_V_7 current that was active at rest in these structures (Fig. 1). Despite this, electrophysiological recordings and computational modeling indicated that the current had little effect on bouton RMP (Fig. 2, Fig. 4). Electrophysiological recordings and computational modeling, though, showed that the K_V_7 current acts as a shunt: restricting *R*_N_ at depolarizing potentials to rest (Figs. 2 and 4). Consequently, K_V_7 currents influenced the probability of spikes elicited with a given depolarization (Fig. 2). Further, K_V_7 currents were found to restrict the spike width and ADP amplitude and decay following a spike (Fig. 3). Computational modeling suggested that this was due to K_V_7 currents opposing Ca^2+^ influx via N- and P/Q-type Ca^2+^ channels during an action potential (Fig. 4). Consistent with this, the presence of either the Ca^2+^ chelator, BAPTA, or the N- and P/Q-type Ca^2+^ channel inhibitors prevented the spike width broadening and the increase in ADP after K_V_7 current inhibition (Figs. 5 and 6). Moreover, in agreement with our premise that K_V_7 currents restrict spike-induced Ca^2+^ influx in mossy fiber boutons, this current significantly reduced action potential-induced neurotransmitter release from mossy fiber boutons and short-term synaptic plasticity at the mossy fiber bouton–CA3 synapse (Fig. 7). These findings indicate that K_V_7 channels play a substantial role in modulating intrinsic excitability and synaptic plasticity at mature synapses in the hippocampus. Our findings also imply that K_V_7 channels are likely to be involved in processes underpinning information storage in the hippocampus.

The mossy fiber bouton K_V_7 current half–maximal activation voltage was more negative (−70 mV, Fig. 1d) than that in hippocampal and neocortical pyramidal neuron somata and axon initial segments^39,58^ but consistent with that at granule cell somata^40^, in peripheral axons^59^ and in Calyx of Held terminals^24^. Hence, ~ 20% of the current was active at rest in mossy fiber bouton terminals. The lack of effect of the current on the bouton RMP is probably owing to the inward rectifier K^+^ current as this has been shown to exert a strong influence on RMP in granule cells^42,43,60^. Unlike in heterologous systems^61^, though, K_V_7 channel inhibitors exert their effects at negative potentials in native cells as they depolarized the RMP and enhanced *R*_N_ in hippocampal and neocortical pyramidal neurons^18,24,39,58,62,63^. Further, axonal K_V_7 currents in peripheral nerve fibers have a XE991-sensitive component at ~ −70 mV^59^. In mossy fiber boutons, consistent with our computational model (Fig. 4a), XE991 increased R_N_ measured using small subthreshold depolarizing pulses in a time-dependent manner too (Fig. 2f).

K_V_7 currents affect mossy fiber bouton terminal excitability distinctly from that of Calyx of Held terminals^24^. In particular, unlike in Calyx of Held terminals, K_V_7 currents regulate the spike width and ADP following spikes in mossy fiber boutons. These effects are unlikely to be due to non-selective effects of K_V_7 channel inhibitors as, at the concentrations utilized, XE991 and linopirdine were at least 50-fold and three fold more potent, respectively, for K_V_7 than other K^+^ channels^64^.

K_V_7 currents also restrict the generation of an intrinsic ADP succeeding spikes in CA1, CA3 and cortical pyramidal neurons^58,62,65–67^. In CA1 and cortical pyramidal neurons, K_V_7 currents counteract a persistent Na^+^ current to reduce the ADP following spikes^58,62,65^. In these neurons, R-type Ca^2+^ tail currents^68^ and Ca^2+^-activated cation channels such as TRPC channels activated via G-protein coupled receptors^40,69–71^ can contribute to the ADP following spikes too. However, whilst hippocampal granule cell somata and dendrites highly express TRPC subunits, immunohistochemistry suggested that TRPC subunits are present intracellularly within mossy fiber axons and boutons^72^. We cannot, though, exclude the possibility of Ca^2+^-activated conductances underlying the ADP generated by K_V_7 current inhibition. Since we do not know if these are present in mossy fiber terminals or their biophysical properties, we were unable to include them in our computational model (see Methods and Supplementary Table 3).

Our computational model included a Ca^2+^ conductance whose decay time constants were slowed down to mimic the Ca^2+^ transient generated in response to a single action potential in a mossy fiber terminal obtained using Ca^2+^ imaging^47–49^. The decay time constant of the Ca^2+^ conductance was considerably slower than that reported for either N-, P/Q-or R-type Ca^2+^ currents in mossy fiber terminals^51^ and may reflect processes such as Ca^2+^-induced Ca^2+^ release^48^ or the effects of endogenous buffers on intracellular Ca^2+^  ^47^. The presence of this Ca^2+^ conductance resulted in an ADP following K_V_7 conductance removal in our simulations (Fig. 4), albeit smaller and faster than that observed following single action potentials under our experimental conditions (Fig. 3). This suggests that, as in CA1 pyramidal neurons^68^, a Ca^2+^ tail current as well as processes such as Ca^2+^-induced Ca^2+^ release may contribute to the ADP generation following K_V_7 current suppression. However, as the simulated ADP amplitude and decay are clearly different from that observed experimentally (Fig. 3), other conductances, such as Ca^2+^-activated conductances, may also contribute to the K_V_7 current inhibition-induced ADP in terminals.

The main physiological role of presynaptic K_V_7 channels in mossy fiber boutons is to regulate synaptic transmission onto CA3 pyramidal neurons (Fig. 7). As spike broadening has been associated with enhanced neurotransmission from synaptic terminals^5,7,14,73^ and K_V_7 current inhibition results in wider action potentials in mossy fiber boutons (Fig. 3), this may have contributed to the greater action potential-induced neurotransmitter release in the absence of K_V_7 currents (Fig. 7). Further, the enhanced ADP resulting from K_V_7 current inhibition is probably due to augmented Ca^2+^ influx and may result in elevated asynchronous release from these terminals. This, though, needs to be further tested. Notwithstanding, the mechanisms by which a reduction in K_V_7 currents in mossy fiber boutons might lead to greater neurotransmitter release differs from that in Calyx of Held terminals, whereby RMP depolarization was largely attributed to the increase in neurotransmission following K_V_7 current block^31^. The effect of K_V_7 currents on intrinsic excitability and synaptic transmission may also vary in different conditions. Indeed, during elevated extracellular K^+^ conditions when axons are already depolarized, these currents in CA3 axons have been suggested to influence Na^+^ current inactivation and increase presynaptic action potential amplitude, Ca^2+^ influx and enhance synaptic release^66^. Thus, K_V_7 currents may have synapse-specific effects on intrinsic excitability which may depend on particular conditions.

Given that K_V_7 currents in mossy fiber boutons significantly augmented the membrane conductance (Figs. 2 and 4), it is probable that, like in Calyx of Held terminals^24^, the current will affect excitatory synaptic potential amplitudes and shapes (i.e., analog signaling) in these terminals. As analog signaling in mossy fiber boutons influences neurotransmission^73^, this might be another mechanism by which K_V_7 currents might regulate synaptic release. Hence, K_V_7 currents might affect neurotransmission by multiple mechanisms, including action potential-dependent release (Fig. 7).

What effect might modulation of neurotransmitter release by K_V_7 channels have on CA3 neural network excitability? Our findings suggest that K_V_7 channels restricts neurotransmitter release from mossy fiber boutons elicited by trains of action potentials occurring at theta and gamma frequencies and will thereby, contribute to maintaining CA3 pyramidal neuronal excitability (Fig. 6). Although the giant mossy fiber bouton–CA3 pyramidal neuron connectivity is sparse (with ~ 50 granule cells contacting one CA3 neuron), each bouton contains an average of 20 release sites^33,74,75^. Thus, changes in K_V_7 channel activity could have an impact on the overall CA3 pyramidal neuron excitability. Indeed, a loss of K_V_7.2 subunits, which are present on mossy fibers and, most likely, their boutons^26–28^, led to impaired hippocampal gamma rhythms and spatial learning as well as spontaneous seizures in rodents, implicating hyperexcitability of cortical neural networks^35^. Thus, the presence of these channels at the mossy fiber bouton, which is a conditional detonator synapse, is likely to be vital for preventing CA3 neuronal and neural circuit hyperactivity. Further, as the K_V_7 current significantly affects short-term synaptic plasticity at the mossy fiber–CA3 synapse (Fig. 6), these channels at this synapse may also affect long-term synaptic plasticity and thus, memory encoding.

## Methods

### Acute slice preparation

The UK Home Office approved all procedures. Hippocampal slices were prepared as described in^76^. In brief, 22–28 day-old male Sprague Dawley rat pups were decapitated, the brain removed and submerged in ice-cold solution (mM): 87 NaCl, 25 NaHCO_3_, 10 glucose, 75 sucrose, 2.5 KCl, 1.25 NaH_2_PO_4_, 0.5 CaCl_2_, 7 MgCl_2_, pH 7.3, 325 mOsm/L. The brain was hemi-sected and a cut parallel to the dorsal part of the brain made. The dorsal side brain halves were glued onto a slice holder and 300–400 μm slices made (Leica VT1200S, Leica, UK). Slices were incubated in the cutting solution for 30–40 min at 35 °C and then stored in the cutting solution at room temperature.

### Electrophysiological recordings

Slices were transferred to a submerged chamber containing external solution (mm): 125 NaCl, 25 NaHCO_3_, 25 glucose, 2.5 KCl, 1.25 NaH_2_PO_4_, 2 CaCl_2_, 1 MgCl_2_, 0.05 CNQX, 0.05 DL-AP5, 0.01 bicuculline, 0.001 CGP 55845, pH 7.3, 32–36 °C. For whole-cell current-clamp recordings, the internal pipette solution contained (mm): 120 KMeSO4, 15 KCl, 10 HEPES, 2 MgCl2, 0.2 EGTA, 2 Na_2_ATP, 0.3 Tris-GTP and 14 Tris-phosphocreatinine, pH 7.3 with KOH, 295–300 mOsm/L. In some experiments, 10 or 20 mM K_4_BAPTA was added to the pipette solution. In this case, the KMeSO_4_ was reduced accordingly to 50 or 60 mm, respectively and osmolarity adjusted by adding *N*-methyl-d-glucamine. Pipettes had resistances of 5–8 MΩ. In all experiments, Neurobiotin (0.2% w/v) was included in the intracellular pipette solution. Slices were fixed in 4% paraformaldehyde and stained with streptavidin Alexa Fluor 488 conjugate (0.04 mg/ml) 24 h later^77^.

Electrophysiological recordings were made using a Multiclamp 700B amplifier (Molecular Devices, UK). Current-clamp recordings were filtered at 10 kHz, and sampled at 50 kHz. Protocols (including that of *R*_N_ (Fig. 2), action potentials initiated with depolarizing steps (Fig. 2) and trains of action potentials (Fig. 3) were applied every minute after application of K_V_7 current inhibitors to facilitate their effects during these recordings^61^. Data were acquired using pClamp 10.0 (Molecular Devices, UK). Series resistance was in the order of 10–30 MΩ. Recordings were discarded if the series resistance increased by > 20%.

*K*_*V*_*7 current recordings*: The external solution was supplemented with 0.001 mm tetrodotoxin and 0.1 mm 4-aminopyridine. The internal solution described above was present in the patch pipette. For outside-out recordings, the whole-cell configuration was first obtained and the patch pipette slowly withdrawn. Series resistance was between 10 and 20 MΩ and was ~ 70% compensated. A de-activation protocol (as described in ref. ^39^; Fig. 1b) was applied in the absence and presence of the K_V_7/M–channel blocker, XE991 (3 μm). Recordings were filtered at 1 kHz and sampled at 10 kHz.

*Paired bouton–CA3 recordings:* Cell-attached recordings from mossy fiber boutons were obtained. The internal solution was as described above. Action currents were elicited in the cell-attached mode by applying 800 mV, 0.1 ms pulses. The internal solution for CA3 neuron whole-cell recordings contained (mm): 135 CsCl, 5 QX314 bromide, 10 HEPES, 2 MgCl2, 0.2 EGTA, 2 Na_2_ATP, 0.3 Tris-GTP and 14 Tris-phosphocreatinine, pH 7.3 with CsOH, 295–300 mOsm/L. Glutamate and GABA receptor blockers were omitted from the external solution. Voltage-clamp recordings were obtained from CA3 pyramidal cells using a Multiclamp 700B amplifier (Molecular Devices, UK). Recordings were filtered at 1 kHz, and sampled at 10 kHz. Post-synaptic series resistance was in the order of 10–20 MΩ. Recordings were discarded if the series resistance increased by > 20%.

All reagents were purchased from Sigma-Aldrich UK apart from tetrodotoxin, bicuculline, CGP 55845, DL-AP5 and XE991, which were obtained from Abcam Ltd (UK). Neurobiotin was acquired from Vector Laboratories Ltd and streptavidin Alexa Fluor 488 was procured from Life Technologies.

### Data analysis

Clampfit (v10.4 or v10.7) was used. To calculate *R*_N_, the difference in steady-state voltage in the last 25 ms elicited by 1 s hyperpolarizing step at − 80 mV was divided by the applied current. Action potentials elicited by 1 s depolarizing steps were counted. Action potential height was measured from threshold to the peak, whereas action potential width was the breadth at half the height. The amplitude and decay time constant of the ADP following single action potentials evoked at 1 or 5 Hz were also measured. To obtain the decay time constant, the decay phase of the ADP was fitted with a double exponential function:

$${\mathrm{ADP}}\,{\mathrm{decay}} = {\mathrm{A}}_{1}{\mathrm{e}}^{\left( { - {{{\mathrm{t}}/\tau 1}}} \right)} + {\mathrm{A}}_{2}{\mathrm{e}}^{\left( { - {{{\mathrm{t}}/\tau 2}}} \right)}$$

where *τ*1 and *τ*2 represent time constants of the initial and falling phase of the ADP. *τ*2 values have been reported in Results and Figures. In addition, the area under the ADP generated during and following a train of action potentials at 20, 50, and 100 Hz was measured before and after application of XE991 (3 μm). There was some variability (albeit non-significant) in these parameters between control recordings from individual mossy fiber boutons (see Fig. 3, Fig. 5, Supplementary Table 1).

For paired bouton–CA3 recordings, the amplitude of the EPSCs generated in response to the action current in the bouton was measured. Both first and second EPSC amplitudes were measured from their directly preceding baselines, respectively. The 10–90% rise time of the EPSC was obtained in Clampfit 10.4 using the function:

$${\mathrm{Slope}} = {\mathrm{A}} \ast 0.8{\mathrm{/}}\left( {{\mathit{t}}2 - {\mathit{t}}1} \right)$$

Where A is the peak amplitude of the EPSC and *t*1 and *t*2 are the times at 10% and 90% of A, respectively. The decay phase of the EPSC was fitted with the above double exponential equation with *τ*1 and *τ*2 represent time constants of the initial and falling phase of the EPSC. Again only *τ*2 values are reported. Paired pulse ratios were calculated as the peak of the second EPSC divided by the peak of the first EPSC.

For K_V_7/M current voltage-clamp data, the traces obtained in the presence of the XE991 (3 μm) were subtracted from those in the absence. The subtracted traces were fitted with the above double exponential function with *τ*1 and *τ*2, representing the de-activation time constants of the initial and late phase of the K_V_7 current.

The K_V_7 conductance values were generated from the normalized amplitudes of the subtracted currents^39^. For whole-cell voltage-clamp experiments, the absolute voltage recorded was subtracted from the estimated reversal potential of K^+^ (E_K_). This together with the current amplitude recorded was used to calculate the conductance and were plotted against the absolute voltage. The curves were fitted using the Boltzmann equation:

$${\mathit{y}} = {\mathrm{A}}2 + \left( {{\mathrm{A}}1 - {\mathrm{A}}2} \right){\mathrm{/}}\left( {1 + {\mathrm{exp}}\left( { - \left( {{\mathit{x}} - {\mathit{x}}0} \right){\mathit{/dx}}} \right)} \right)$$

where A1 and A2 are the initial and maximum values, *x*0 is the half-activation voltage and *dx* is the slope of the curve.

### Statistical analysis

Group data are expressed as mean ± SEM. In all experiments, a minimum of three brain slice preparations made from three independent animals were used. For experiments involving pharmacological drug application (i.e., XE991, linopirdine or N- and P/Q-type voltage-gated Ca^2+^ channel inhibitors), paired *t* tests were used with statistical significance determined to be *p* < 0.05. Significant differences at *p* < 0.05 is indicated as asterisks (*) in all figures.

### Computational modeling

All simulations were carried out using the NEURON simulation environment (v7.5)^78^. All model and simulation files will be uploaded to the ModelDB database (https://senselab.med.yale.edu/modeldb/ accession no. 245417). The mossy fiber synaptic bouton was modeled as a single compartment (length = 3.5 μm, diameter = 2 μm, Cm = 1 μF/cm^2^, *R*m = 30 kΩ/cm^2^, *R*a = 150 Ω cm). Temperature was set at 34 °C. Active properties included a transient Na^+^ conductance, four types of K^+^ currents (delayed rectifier type K^+^ conductance, A-type K^+^ conductance, K_V_7 conductance, and inward rectifier type K^+^ conductance), a Ca^2+^ conductance (which is owing to all Ca^2+^ conductances in the bouton including N-, P/Q- and R-type Ca^2+^ conductances), and a simple Ca^2+^-extrusion mechanism with a 500 ms time constant, which is consistent with that reported by other studies^47–49^. Kinetics for the delayed rectifier type K^+^ conductance, A-type K^+^ conductance and Ca^2+^ conductance were taken from a previously published model^39^ (ModelDB accession no. 112546); the Na^+^ kinetics was implemented as in ref. ^44^; the inward rectifier K^+^ conductance was implemented as in ref. ^60^ (downloaded from ModelDB, accession no. 185355). The peak conductances used in all simulations and kinetic parameters modified with respect to their original values are reported in Supplementary Table 3. Increasing the peak conductance of the delayed rectifier conductance had little effect on the simulations. The Ca^2+^ conductance peak value and decay time constant were adjusted to reflect Ca^2+^ transient measured in response to an action potential in a mossy fiber bouton. The effects of XE991 application were modeled with a complete block of the K_V_7 conductance. A single action potential was elicited with a current pulse of 30 pA for 0.35 ms. To simulate a 50 Hz stimulation, the model was stimulated every 20 ms with 30 pA 0.3 ms long current pulses. Input resistance was measured from the voltage deflection caused by a 1 s long 0.3 pA current injection.

### Reporting summary

Further information on research design is available in the Nature Research Reporting Summary linked to this article.

## Supplementary information

Supplementary Information

Reporting Summary

## Data Availability

All experimental data generated or analyzed during this study are included in this article, supplementary information files, and is available from the authors upon reasonable request.

## Code availability

The complete set of files for the computational model are available on ModelDB (https://senselab.med.yale.edu/modeldb/ accession no. 245417).
