# Supplementary material for: The subthreshold-active KV7 current regulates neurotransmission by limiting spike-induced Ca2+ influx in hippocampal mossy fiber synaptic terminals
Source: Commun Biol. 2019 Apr 26;2:145. doi: 10.1038/s42003-019-0408-4 (PMC6486593; doi:10.1038/s42003-019-0408-4)
Supplement: Supplementary file 1 — Supplementary Information [file 42003_2019_408_MOESM1_ESM.pdf]

Supplementary Figure 1

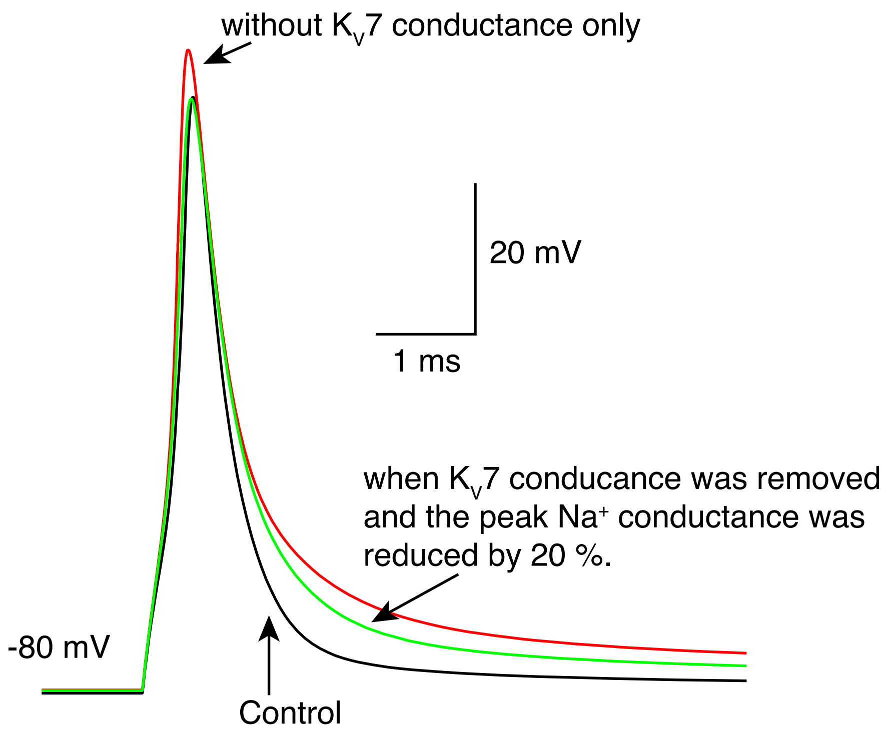

Supplementary Fig 1: A simulated single action potential elicited with a 30 pA, 0.35 ms current injection under control conditions (black) and when the K_V7 conductance was removed (red). The action potential amplitude when the K_V7 conductance was removed was enhanced and this could be restored to control conditions by reducing the peak Na^+ conductance by 20 % as illustrated in green.

Supplementary Table 1.

	ADP Amplitude (mV)		ADP decay time constant (ms)	
	1st AP	20th AP	1st AP	20th AP
Control 1 Hz	6.49 ± 1.03 (n=8)	6.72 ± 0.93 (n=8)	66.43 ± 6.54 (n=7)	67.71 ± 5.92 (n=7)
+XE991 1 Hz	10.44 ± 1.18* (n=8)	10.18 ± 0.96* (n=8)	115.43 ± 10.75* (n=7)	112.86 ± 9.03* (n=7)
Control 1 Hz	9.90 ± 1.30 (n=4)	9.33 ± 1.79 (n=4)	51.49 ± 15.14 (n=3)	44.64 ± 9.99 (n=4)
+Linopirdine 1 Hz	15.42 ± 2.11* (n=4)	17.73 ± 3.75* (n=4)	69.50 ± 17.53* (n=4)	68.03 ± 18.57* (n=4)
Control 5 Hz	6.52 ± 0.83 (n=8)	6.37 ± 0.84 (n=8)	58.14 ± 6.31 (n=7)	55.14 ± 5.57 (n=7)
+XE991 5 Hz	9.79 ± 1.09* (n=8)	9.18 ± 1.13* (n=8)	107.43 ± 9.52* (n=7)	105.00 ± 9.05* (n=7)
Control 5 Hz	9.84 ± 1.03 (n=4)	12.58 ± 1.28 (n=4)	28.77 ± 8.24 (n=4)	38.72 ± 12.96 (n=4)
+Linopirdine 5 Hz	19.69 ± 2.25* (n=4)	20.56 ± 2.35* (n=4)	61.92 ± 20.62* (n=4)	60.24 ± 17.39* (n=4)
BAPTA 1 Hz	1.57 ± 1.21 (n=11)	1.72 ± 1.44 (n=11)	35.36 ± 21.70 (n=11)	36.64 ± 22.56 (n=11)
+XE991 1 Hz	1.89 ± 2.14 (n=11)	1.94 ± 2.67 (n=11)	32.27 ± 21.34 (n=11)	31.82 ± 21.73 (n=11)
BAPTA 5 Hz	1.88 ± 1.80 (n=11)	1.84 ± 1.78 (n=11)	35.73 ± 25.19 (n=11)	37.09 ± 25.50 (n=11)
+XE991 5 Hz	1.74 ± 1.71 (n=11)	1.94 ± 1.87 (n=11)	31.27 ± 24.31 (n=11)	31.64 ± 25.60 (n=11)
Control 1 Hz	9.72 ± 0.66 (n=6)	10.14 ± 0.82 (n=6)	98.17 ± 20.54 (n=6)	111.00 ± 22.44 (n=6)
+XE991 and N/P/Q Ca^{2+} channel inhibitors 1 Hz	3.85 ± 0.62* (n=6)	3.92 ± 0.70* (n=6)	45.67 ± 6.20* (n=6)	43.00 ± 3.94* (n=6)
Control 5 Hz	10.26 ± 0.75 (n=6)	10.55 ± 1.08 (n=6)	94.67 ± 20.14 (n=6)	114.33 ± 24.02 (n=6)
+XE991 and N/P/Q Ca^{2+} channel inhibitors 5 Hz	4.17 ± 0.58* (n=6)	4.10 ± 0.40* (n=6)	47.17 ± 5.82* (n=6)	49.17 ± 6.17* (n=6)

Supplementary Table 1: The mean and standard error of mean values for the amplitude and decay time constant of the ADP following either the 1st or 20th action potential (AP) in train elicited at 1 Hz or 5 Hz under control conditions or when BAPTA (10 mM or 20 mM) was incorporated in the patch pipette and after subsequent application of 3 μM XE991 or 10 μM Linopirdine or 3 μM XE991 together with 100 nM ω-conotoxin GVIA and 100 nM ω-agatoxin IVA. The numbers of observations are shown in parenthesis. Asterisks (*) indicate significance at $p < 0.05$ when compared with the appropriate controls.

Supplementary Table 2.

	AP Amplitude (mV)		AP Half-width (ms)	
	1st AP	20th AP	1st AP	20th AP
Control 1 Hz (n=8)	65.52 ± 4.18	64.59 ± 3.00	0.85 ± 0.05	0.86 ± 0.05
+XE991 1 Hz (n=8)	57.47 ± 2.92*	58.60 ± 3.89*	1.04 ± 0.10*	1.09 ± 0.10*
Control 1 Hz (n=4)	60.26 ± 2.33	63.21 ± 2.64	1.23 ± 0.13	1.24 ± 0.13
+Linopirdine 1 Hz (n=4)	49.68 ± 0.83*	48.82 ± 1.80*	1.37 ± 0.17*	1.36 ± 0.15*
Control 5 Hz (n=8)	66.31 ± 2.27	61.02 ± 2.35	0.85 ± 0.05	0.97 ± 0.05
+XE991 5 Hz (n=8)	58.17 ± 2.66*	55.73 ± 2.25*	1.07 ± 0.10*	1.18 ± 0.09*
Control 5 Hz (n=4)	60.71 ± 3.60	61.87 ± 3.51	1.24 ± 0.12	1.28 ± 0.13
+Linopirdine 5 Hz (n=4)	50.20 ± 2.19*	46.68 ± 0.98*	1.39 ± 0.15*	1.44 ± 0.17*
Control 20 Hz (n=8)	63.20 ± 2.64	60.10 ± 2.67	0.83 ± 0.05	0.99 ± 0.05
+XE991 20 Hz (n=8)	56.70 ± 1.58*	53.53 ± 2.21*	1.08 ± 0.10*	1.16 ± 0.09*
Control 20 Hz (n=4)	64.37 ± 2.73	61.10 ± 4.36	1.30 ± 0.13	1.39 ± 0.11
+Linopirdine 20 Hz (n=4)	46.72 ± 1.20*	43.69 ± 4.07*	1.46 ± 0.13	1.67 ± 0.17
Control 50 Hz (n=8)	66.87 ± 3.18	55.56 ± 3.22	1.07 ± 0.11	1.13 ± 0.07
+XE991 50 Hz (n=8)	60.01 ± 2.66*	50.57 ± 3.01*	1.26 ± 0.07*	1.50 ± 0.07*
Control 50 Hz (n=4)	60.90 ± 1.59	58.03 ± 2.74	1.29 ± 0.14	1.60 ± 0.16
+Linopirdine 50 Hz (n=4)	46.97 ± 3.87*	42.50 ± 1.75*	1.50 ± 0.14*	1.77 ± 0.20*
Control 100 Hz (n=8)	63.86 ± 3.20	51.74 ± 3.13	0.79 ± 0.04	1.08 ± 0.05
+XE991 100 Hz (n=8)	58.10 ± 3.22*	43.34 ± 2.03*	1.00 ± 0.09*	1.22 ± 0.08*
Control 100 Hz (n=4)	61.64 ± 2.07	55.73 ± 4.28	1.30 ± 0.13	1.43 ± 0.13
+Linopirdine 100 Hz (n=4)	47.51 ± 0.98*	35.92 ± 3.44*	1.51 ± 0.13*	1.85 ± 0.17*
BAPTA 1 Hz (n=11)	91.18 ± 2.91	90.36 ± 3.03	2.45 ± 0.23	4.32 ± 1.58
+XE991 1 Hz (n=11)	91.45 ± 3.13	91.21 ± 3.43	2.65 ± 0.27	4.52 ± 1.57
BAPTA 5 Hz (n=11)	90.18 ± 2.78	88.91 ± 2.96	2.53 ± 0.24	4.50 ± 1.56

+XE991 5 Hz (n=11)	91.63 ± 3.11	90.18 ± 3.32	2.74 ± 0.28	4.81 ± 1.54
BAPTA 20 Hz (n=11)	90.91 ± 2.68	85.64 ± 2.71	2.53 ± 0.25	4.75 ± 1.54
+XE991 20 Hz (n=11)	91.18 ± 2.79	86.09 ± 2.36	2.72 ± 0.26	4.96 ± 1.52
BAPTA 50 Hz (n=11)	90.68 ± 2.44	82.14 ± 2.41	2.37 ± 0.22	4.68 ± 1.54
+XE991 50 Hz (n=11)	91.09 ± 2.48	79.22 ± 2.25	2.77 ± 0.31	4.95 ± 1.52
BAPTA 100 Hz (n=11)	90.45 ± 3.15	63.82 ± 3.55	2.40 ± 0.22	4.74 ± 1.54
+XE991 100 Hz (n=11)	88.45 ± 3.62	65.36 ± 3.00	2.64 ± 0.29	4.85 ± 1.53
Control 1 Hz (n=4)	83.25 ± 6.80	82.75 ± 6.55	2.48 ± 0.38	2.88 ± 0.24
+XE991 and N/P/Q Ca^{2+} channel inhibitors 1 Hz (n=4)	76.75 ± 6.37*	74.50 ± 7.08*	3.10 ± 0.39	2.85 ± 0.68
Control 5 Hz (n=6)	94.67 ± 3.97	93.00 ± 3.55	2.30 ± 0.28	2.70 ± 0.28
+XE991 and N/P/Q Ca^{2+} channel inhibitors 5 Hz (n=6)	89.00 ± 3.72*	87.33 ± 3.31*	2.48 ± 0.24	2.87 ± 0.24
Control 20 Hz (n=6)	92.83 ± 3.38	85.86 ± 5.45	2.40 ± 0.24	2.83 ± 0.36
+XE991 and N/P/Q Ca^{2+} channel inhibitors 20 Hz (n=6)	81.67 ± 6.23*	77.06 ± 6.57*	2.88 ± 0.32	3.28 ± 0.28
Control 50 Hz (n=6)	86.50 ± 5.62	76.83 ± 6.11	2.68 ± 0.37	3.08 ± 0.36
+XE991 and N/P/Q Ca^{2+} channel inhibitors 50 Hz (n=6)	68.52 ± 5.69*	62.05 ± 5.12*	3.1 ± 0.29	3.87 ± 0.31
Control 100 Hz (n=5)	88.00 ± 5.17	67.20 ± 5.54	2.36 ± 0.32	3.22 ± 0.38
+XE991 and N/P/Q Ca^{2+} channel inhibitors 100 Hz (n=5)	64.47 ± 9.22*	59.20 ± 5.08*	2.70 ± 0.30	3.60 ± 0.36

Supplementary Table 2: The mean and standard error of mean values for the amplitude and half-width of the 1st and 20th action potential (AP) in a train elicited at frequencies ranging from 1 Hz – 100 Hz under control conditions or when BAPTA (10 mM or 20 mM) was incorporated in the patch pipette and after subsequent application of 3 μM XE991 or 10 μM Linopirdine or 3 μM XE991 together with 100 nM ω-conotoxin GVIA and 100 nM ω-agatoxin IVA. The numbers of observations per group are shown in parenthesis. Asterisks (*) indicate significance at $p < 0.05$ when compared with the appropriate controls.

Supplementary Table 3.

Conductance	Peak Conductance (S/cm^2)	$V_{1/2}$ activation (mV)	$V_{1/2}$ inactivation (mV)	Reference
Na^+	0.12	-40.4	-86.8	Engel and Jonas (2005)[44]
Delayed rectifier type K^+	0.001	13		Shah et al., (2008)[39]
A-type K^+	0.01	11	-56	Shah et al., (2008)[39]
Inward rectifier type K^+	0.021	-98.92		Stegen et al., (2012)[57]
K_v7	0.005	-70		Data from this manuscript
Ca^{2+}	0.6	-14	-40.11	Shah et al., (2008)[39]

Supplementary Table 3: Selected properties of the conductances used in our computational model. The complete set of files are available on ModelDB (https://senselab.med.yale.edu/modeldb/ accession no. 245417). The Na^+ conductance kinetics was implemented using the fitting parameters reported in Engel and Jonas (2005)[44]. The deactivation time constant of the Ca^{2+} conductance was set to 50 ms. For experimental values that were obtained at room temperature, a Q10 value of 5 was used.
