# Supplementary material for: The subthreshold-active KV7 current regulates neurotransmission by limiting spike-induced Ca2+ influx in hippocampal mossy fiber synaptic terminals
Source: Commun Biol. 2019 Apr 26;2:145. doi: 10.1038/s42003-019-0408-4 (PMC6486593; doi:10.1038/s42003-019-0408-4)
Supplement: Supplementary file 2 — Reporting Summary [file 42003_2019_408_MOESM2_ESM.pdf]

nature research

Corresponding author(s): Mala M. Shah

Last updated by author(s): Mar 14, 2019

Reporting Summary

Nature Research wishes to improve the reproducibility of the work that we publish. This form provides structure for consistency and transparency in reporting. For further information on Nature Research policies, see Authors & Referees and the Editorial Policy Checklist.

Statistics

For all statistical analyses, confirm that the following items are present in the figure legend, table legend, main text, or Methods section.

n/a	Confirmed	
☐	☒	The exact sample size (*n*) for each experimental group/condition, given as a discrete number and unit of measurement
☐	☒	A statement on whether measurements were taken from distinct samples or whether the same sample was measured repeatedly
☐	☒	The statistical test(s) used AND whether they are one- or two-sided<br>*Only common tests should be described solely by name; describe more complex techniques in the Methods section.*
☒	☐	A description of all covariates tested
☒	☐	A description of any assumptions or corrections, such as tests of normality and adjustment for multiple comparisons
☐	☒	A full description of the statistical parameters including central tendency (e.g. means) or other basic estimates (e.g. regression coefficient) AND variation (e.g. standard deviation) or associated estimates of uncertainty (e.g. confidence intervals)
☒	☐	For null hypothesis testing, the test statistic (e.g. *F*, *t*, *r*) with confidence intervals, effect sizes, degrees of freedom and *P* value noted<br>*Give P values as exact values whenever suitable.*
☒	☐	For Bayesian analysis, information on the choice of priors and Markov chain Monte Carlo settings
☒	☐	For hierarchical and complex designs, identification of the appropriate level for tests and full reporting of outcomes
☒	☐	Estimates of effect sizes (e.g. Cohen's *d*, Pearson's *r*), indicating how they were calculated

Our web collection on statistics for biologists contains articles on many of the points above.

Software and code

Policy information about availability of computer code

Data collection	For acquisition of electrophysiological data, we used commercially available software (PClamp, molecular Devices) as stated in the Methods Section (Pg 18).<br>For computational modelling, we used the Neuron simulation environment (Pg 21; again this is commercially available and free to download).
Data analysis	We used Clampfit (commercially available) to analyse raw electrophysiological data. We then further analysed the data using Excel.<br>For data fitting, Clampfit or Origin Pro 9.1 were used.

For manuscripts utilizing custom algorithms or software that are central to the research but not yet described in published literature, software must be made available to editors/reviewers. We strongly encourage code deposition in a community repository (e.g. GitHub). See the Nature Research guidelines for submitting code & software for further information.

Data

Policy information about availability of data

All manuscripts must include a data availability statement. This statement should provide the following information, where applicable:

- Accession codes, unique identifiers, or web links for publicly available datasets
- A list of figures that have associated raw data
- A description of any restrictions on data availability

All experimental data generated or analysed during this study are included in this article(Figs 1, 2, 3, 5, 6, 7) and it supplementary information files (Supp Fig 1, Supp Table 1-3).
As stated on Page 21, all model and simulation files will be uploaded to the ModelDB database (https://senselab.med.yale.edu/modeldb/ accession no. 245417).

Field-specific reporting

Please select the one below that is the best fit for your research. If you are not sure, read the appropriate sections before making your selection.

☒ Life sciences ☐ Behavioural & social sciences ☐ Ecological, evolutionary & environmental sciences

For a reference copy of the document with all sections, see nature.com/documents/nr-reporting-summary-flat.pdf

Life sciences study design

All studies must disclose on these points even when the disclosure is negative.

Sample size	No sample size calculation was performed. Each data set was deemed to be complete if it was clear that there was statistical significance or not.
Data exclusions	No data were excluded
Replication	All attempts at replication of data were successful.
Randomization	This was not relevant to the group as the samples were prepared in a similar manner for all experiments. Only the drug applied varied between experiments.
Blinding	The experiments were all pharmacology experiments and computational modelling. The probability of obtaining a good electrophysiological recording from a mossy fiber bouton was approximately once every 5 preparations. The probability of obtaining a dual mossy fiber bouton and CA3 pyramidal neuron recording was once every two months. Given the challenging nature of these experiments, we were trying to make the most of all recordings that we obtained and thus, the experimenter was not blind to the treatment applied.

Reporting for specific materials, systems and methods

We require information from authors about some types of materials, experimental systems and methods used in many studies. Here, indicate whether each material, system or method listed is relevant to your study. If you are not sure if a list item applies to your research, read the appropriate section before selecting a response.

Materials & experimental systems

n/a	Involved in the study	
☐	☒	Antibodies
☒	☐	Eukaryotic cell lines
☒	☐	Palaeontology
☐	☒	Animals and other organisms
☒	☐	Human research participants
☒	☐	Clinical data

Methods

n/a	Involved in the study	
☒	☐	ChIP-seq
☒	☐	Flow cytometry
☒	☐	MRI-based neuroimaging

Antibodies

Antibodies used	streptavidin Alexa Fluor 488 conjugate
Validation	Characterised by Alberran et al., 2005, Protein Eng Des Sel, 18, 147-52; see https://www.thermofisher.com/order/catalog/product/S32354

Animals and other organisms

Policy information about studies involving animals; ARRIVE guidelines recommended for reporting animal research

Laboratory animals	As stated on Pg 17, 22-28 day old male sprague dawley rat pups were used.
Wild animals	The study did not involve wild animals
Field-collected samples	Animals were bred at UCL Central Biological Service Unit. The mother and 14-15 day old pups were transported to School of Pharmacy Biological Service Unit where they were maintained in standard cages with bedding, nesting material and toys such as plastic tunnels. The animals were exposed to a 12 hr light/dark cycle and had 24 hr access to food and water. Animals were maintained for at least 7 days at School of Pharmacy prior to the experiment. For each experiment, as stated on Pg 17, 22 - 28 day old pups were decapitated and the brain rapidly removed. Once all pups were used for experiments, the mother was humanely culled using Schedule 1 methods.

Ethics oversight | The project was approved by the UK Home Office as well as UCL Ethics Committee (see Pg 17)

Note that full information on the approval of the study protocol must also be provided in the manuscript.
